# Critical Role of Tumor Microenvironment in Shaping NK Cell Functions: Implication of Hypoxic Stress

**DOI:** 10.3389/fimmu.2015.00482

**Published:** 2015-09-23

**Authors:** Meriem Hasmim, Yosra Messai, Linda Ziani, Jerome Thiery, Jean-Henri Bouhris, Muhammad Zaeem Noman, Salem Chouaib

**Affiliations:** ^1^INSERM U 1186, Equipe labellisée Ligue Contre le Cancer, Gustave Roussy Campus, Villejuif, France; ^2^Department of Hematology and Bone Marrow Transplantation, Gustave Roussy Campus, Villejuif, France

**Keywords:** HIF, microenvironment, solid tumors, natural killer cells, immune suppression

## Abstract

Blurring the boundary between innate and adaptive immune system, natural killer (NK) cells, a key component of the innate immunity, are recognized as potent anticancer mediators. Extensive studies have been detailed on how NK cells get activated and recognize cancer cells. In contrast, few studies have been focused on how tumor microenvironment-mediated immunosubversion and immunoselection of tumor-resistant variants may impair NK cell function. Accumulating evidences indicate that several cell subsets (macrophages, myeloid-derived suppressive cells, T regulatory cells, dendritic cells, cancer-associated fibroblasts, and tumor cells), their secreted factors, as well as metabolic components (i.e., hypoxia) have immunosuppressive roles in the tumor microenvironment and are able to condition NK cells to become anergic. In this review, we will describe how NK cells react with different stromal cells in the tumor microenvironment. This will be followed by a discussion on the role of hypoxic stress in the regulation of NK cell functions. The aim of this review is to provide a better understanding of how the tumor microenvironment impairs NK cell functions, thereby limiting the use of NK cell-based therapy, and we will attempt to suggest more efficient tools to establish a more favorable tumor microenvironment to boost NK cell cytotoxicity and control tumor progression.

## Introduction

Natural killer (NK) cells are lymphoid cells that are considered to be major innate effector cells. They are endowed with a natural ability to kill tumor cells and infected cells (1). NK cell lytic functions are regulated by a balance of activating and inhibiting signals originating from membrane receptors (1). Despite their effective antitumor activity, their contribution in controlling solid tumor progression remains elusive. The immunosuppressive tumor microenvironment is undoubtedly involved in tumor evasion from NK cell-mediated killing through several cellular and metabolic factors. Immune and stromal cells as well as the hypoxic stress inside the tumor microenvironment are known to be negative regulators of NK cell infiltration into solid tumors and cytotoxicity (1). Tumor cells themselves develop several strategies to evade NK cell-mediated killing. In this regard, hypoxic stress through its ability to induce tumor resistance and to regulate the differentiation and function of immune-suppressive cells plays a determinant role in shaping the NK cell phenotype and function.

In this review, we propose an insight on how tumor microenvironment inhibits NK cell functions and how this may impact the therapeutic use of NK cells in anticancer treatments.

## Cell-Mediated Immune Suppression toward NK Cells in the Tumor Microenvironment

The tumor microenvironment is a complex network of tumor cells, immune cells, stromal cells, and extracellular matrix accomplishing proliferation, migration, and dissemination of tumor cells. The immune cell subset comprises CD8^+^ T cells, CD4^+^ T cells, NK cells, and myeloid cells [dendritic cells (DCs), M2-macrophages, myeloid-derived suppressor cells (MDSCs)]. Despite the recognized role of NK cells in clearing circulating tumor cells (leukemia cells, metastatic cells) (2–4), the antitumor functions of NK cells in solid tumors are frequently mentioned due to the more favorable prognosis associated with higher NK cell infiltration in some type of cancers (5), the inverse correlation between natural cytotoxic activity and cancer incidence (6), or the faster tumor growth in NK cell-depleted mouse models (7–9).

**Figure 1:**
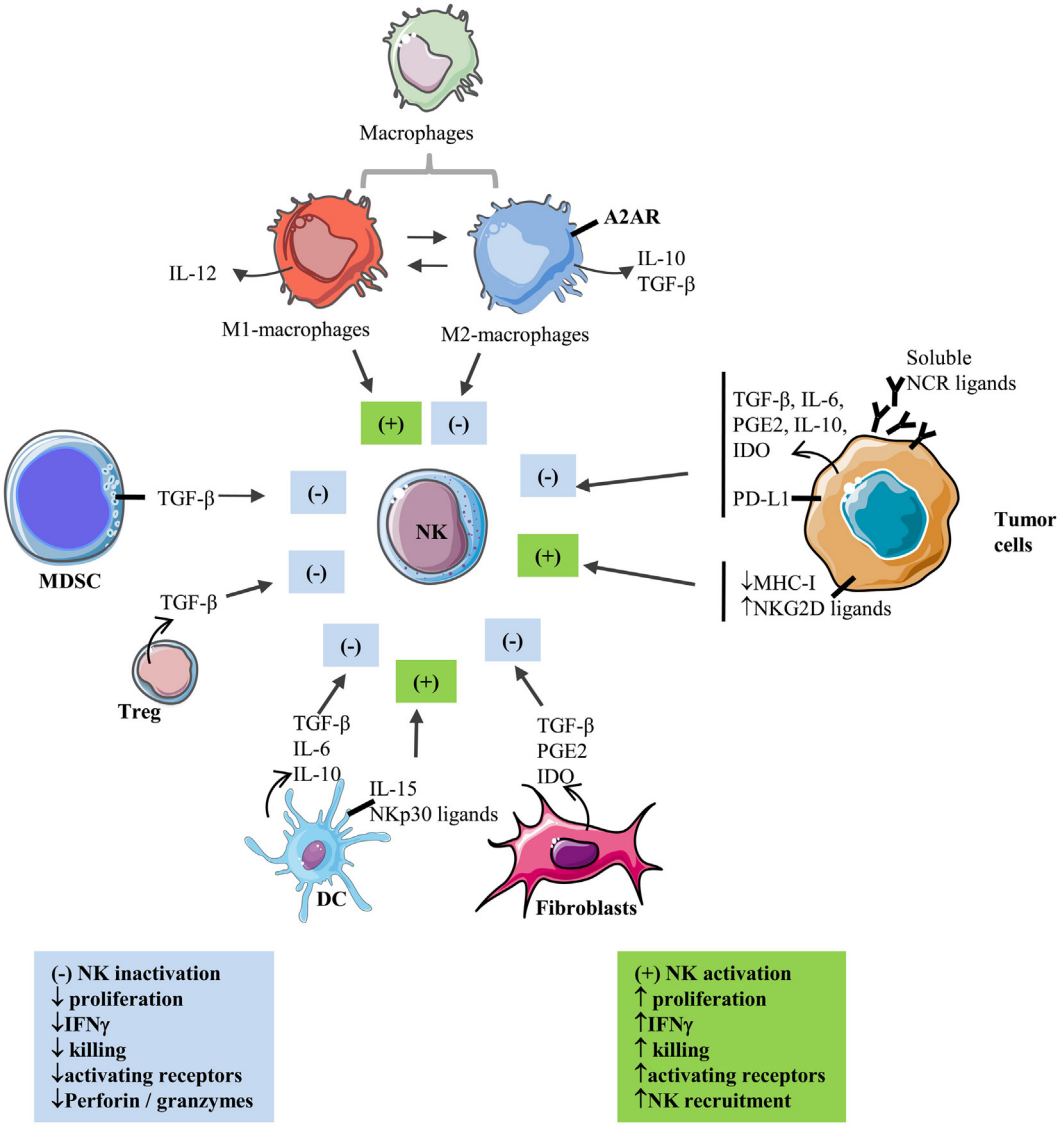
**Interactions between NK and stromal cells within the solid tumor microenvironment**. Activating and inhibiting interactions of stromal cells with NK cells in the tumor microenvironment. 
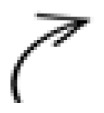
 : secretion; ▬ : membrane-bound; (+): activation; (−): inhibition.

Natural killer cells enter solid tumor site by extravasations through tumor vasculature (10). CXCR3 is a major chemokine receptor involved in NK cell migration toward tumor following a gradient of the tumor-derived chemokine (C-X-C motif) ligands CXCL9, 10, and 11 (11, 12). In particular, increased CXCL10 expression in melanoma tumors results in increased infiltration of adoptively transferred CXCR3-positive expanded NK cells, reflecting the role of CXCL10-induced chemoattraction (12). However, infiltrated NK cells often display a suppressed phenotype inside solid tumors. Accumulating evidence indicates that tumor-residing cells as well as a series of microenvironmental factors are endowed with suppressive properties that affect NK cell reactivity and inhibit their functions (Figure 1).

### Macrophage polarization regulates NK cell-mediated cytotoxicity

Within the tumoral tissue, macrophages and other myeloid cells constitute a major component of the immune infiltrate (13, 14). They differentiate into tumor-associated macrophages (TAM) with expression of TAM markers such as CD206 (15). Exposure of TAM to tumor-derived cytokines such as IL-4, IL-10, IL-13, and M-CSF is able to convert them into polarized type II or M2 macrophages with immune-suppressive activities resulting in tumor progression (15). M2-polarized macrophages appear to contribute to immune suppression through the production of immunosuppressive factors such as IL-10 and TGF-β (16). Recently, the role of myeloid cells including macrophages in immunosuppression of NK cells has been better understood by the involvement of A2AR receptors (17). Myeloid-selective deletion of A2ARs significantly activates macrophages by favoring M1 polarization, reduces lung metastasis, and increases CD44 expression on tumor-associated NK cells and T cells as well as numbers and activation of NK cells and antigen-specific CD8^+^ T cells in lung infiltrates (17). In a xenografted lung carcinoma model, increased expression of surfactant protein-A (SP-A) was reported to be associated with reduced tumor growth and increased M1-TAM and NK cell recruitment and activation at the tumor site (18).

### Myeloid-derived suppressor cells suppress NK cell activity

Myeloid-derived suppressor cells represent additional myeloid subsets involved in tumor-induced immunosuppression (19). MDSCs comprise immature macrophages, granulocytes, and DCs. Their expansion and immunosuppressive functions are well documented in tumor-bearing mice and cancer patients. As such, the NK cell activity was found to be inversely correlated with MDSC expansion (20, 21). In addition, MDSC-mediated inhibition of NK cells was found to be cell contact dependent via membrane-bound transforming growth factor-β (TGF-β) on MDSC (21) or inhibition of perforin and signal transducer and activator of transcription 5 (Stat5) activity in NK cells (20). MDSC from patients with hepatocarcinoma also show inhibitory effects on autologous NK cells after coculture (22). This inhibition was also found to be cell contact dependent and to involve blocking of the activating receptor NKp30 on NK cells (22).

### CD4^+^CD25^+^ T regulatory cells inhibit NK cell cytolytic functions

T regulatory cells (Treg) are well described for their immunosuppressive functions (23). Studies performed by Trzonkowski et al. (24) and Xu et al. (25) report direct inhibitory effects of Treg on NK cell cytolytic functions and expression of the CD69 activation marker following *in vitro* cocultures. These studies indicate that the production of TGF-β by Treg is at least one mechanism of Treg-mediated NK cell inhibition. *In vivo*, Treg depletion was shown to increase NK cell proliferation by a mechanism involving IL-15Rα expression on DCs (26). In a murine model, such depletion was also shown to favor NKG2D-mediated tumor rejection (27).

### Dendritic cells modulate NK cell cytotoxicity

The incrimination of TGF-β in the modulation of NK cell cytotoxicity is also reported when NK cells are cocultured with DCs. Signal transducer and activator of transcription 3 (STAT3) phosphorylation in DCs was reported to be associated with increased secretion of TGF-β, which inhibited NK cell activity, and inhibition of TGF-β restored NK cell functions (28). TGF-β production by DCs can be induced by coculture of immature DC with lung carcinoma cells (29) or by stimulation with LPS (30). Secretion of IL-6 and IL-10 by DC has also been incriminated in dendritic cell-mediated NK cell inhibition (31). Nevertheless, some reports show that DC can also activate NK cell functions. IL-15-stimulated DCs acquire the ability to increase surface expression of the NK cell-activating receptors NKp30 and NKp46, which is associated with an increased tumor target killing (32). This activation is cell contact dependent and required membrane-bound IL-15 on DC. DCs were also reported to induce NK cell proliferation and to activate NKp30 receptor-signaling in NK cells (33).

### Cancer-associated fibroblasts decrease NK cell-mediated cytotoxicity

Among the stromal cells, modified/activated fibroblasts, often termed cancer-associated fibroblasts (CAFs), are considered to play a central role in the complex process of tumor–stroma interaction. CAFs, the prominent stromal cell population in most types of human carcinomas, are α-SMA (alpha-smooth muscle actin) positive, spindle-shaped cells, which closely resemble normal myofibroblasts but express specific markers [i.e., FAP (fibroblast-associated protein), FSP-1 (fibroblast specific protein 1), and PDGFR-β (platelet-derived growth factor)] together with vimentin (a mesenchymal marker) and the absence of epithelial (cytokeratin, E-cadherin) and fully differentiated smooth muscle (smoothelin) markers (34–36). CAFs differentiate in the tumor microenvironment in a TGF-β-dependent manner from other cell types such as resident fibroblasts, mesenchymal stem cells, and endothelial and epithelial cells (37, 38). In the tumor stroma, CAFs produce and secrete several factors such as extracellular matrix proteins (i.e., collagen I, III, IV), matrix metalloproteinases (MMPs), proteoglycans (i.e., laminin, fibronectin), chemokines (i.e., CXCL1, CXCL2, CXCL8, CXCL6, CXCL12/SDF1, CCL2, and CCL5), vascularization promoting factors (i.e., PDGF and VEGF), and other proteins that affect tumor cells’ proliferation, invasiveness, and survival (i.e., TGF-β, EGF, HGF, and FGF) (39). Consequently, CAFs have been involved in tumor growth, angiogenesis, tissue invasion, and metastasis (40).

During the past few years, these activated tumor-associated fibroblasts have also been involved in the modulation of the antitumor immune response, especially by the secretion of soluble immunosuppressive factors in the tumor microenvironment (TGF-β, IL-1β, IL-6, and IL-10) (41). As such, CAFs can potentially affect both innate and adaptive antitumor immune response by increasing the recruitment to tumor of myeloid-derived suppressive cells (MDSC), by decreasing antigen presentation, by increasing the numbers of Tregs, by decreasing T-cell proliferation, cytotoxic T-cell (CTL) function and maturation, or by inhibiting B cell activation and differentiation (41, 42). Different studies also involved CAFs in the modulation of the NK cell functionality. Indeed, the secretion of TGF-β by CAFs could attenuate the expression of NK cell-activating receptors including NKG2D, NKp30, and NKp44 (43, 44). More recently, studies involving melanoma and hepatocellular and colorectal carcinoma-derived fibroblasts have shown that CAFs can decrease NKG2D expression on NK cells surface through the secretion of prostaglandin E2 (PGE2) and/or indoleamine-2,3-dioxygenase (IDO). In parallel, perforin and granzyme B expression (involved in NK cell-mediated killing of target cells) also seem to be decreased by coculture of NK cells with CAFs, affecting their lytic potential (45–47). Altogether, these findings highlight the direct and indirect action of CAFs on various levels of the antitumor immune response and on NK cells antitumor activity within the tumor microenvironment.

### Tumor cells may develop strategies to evade NK cell-mediated lysis

In many cancers, tumor cells down-regulate surface expression of MHC-I molecules in order to evade CD8-dependent T-cell killing, making them more susceptible to NK cell-dependent killing. Cancer cells can also up-regulate NKG2D ligands following activation of NFκB or Sp transcription factors (48). However, tumor cells also develop various strategies to inhibit NK cell-mediated cytotoxicity. Indeed, NK cells from multiple myeloma patients were shown to constitutively express the inhibitory receptor PD-1, as compared to NK cells from healthy donors, which contributes to NK cell inhibition by multiple myeloma cells (49). When the authors inhibit the PD-1/PD-L1 axis using lenalidomide or a blocking antibody, they restored NK cell lytic functions against tumor cells. In addition, tumor cells secrete a number of immunosuppressive cytokines such as TGF-β. In this regard, neuroblastoma cell-derived TGF-β has been reported to down-regulate the activating receptor NKp30 (50). Melanoma cells are also able to inhibit the expression of activating NK cell receptors including NKp30, NKp44, and NKG2D, resulting in impairment of NK cell-mediated cytotoxicity (51). This inhibitory effect is mediated via the production of IDO and PGE2 by melanoma cells. Tumor cells may release soluble NKG2D ligands through proteolytic cleavage, resulting in down-regulation of NKG2D and impairment of NK cell lytic functions (52, 53). The inhibitory consequences of releasing soluble NK cell receptor ligands may not be systematic. Indeed, NKp30 activation by tumor-released vesicles containing HLA-B-associated transcript 3 (BAT3), a ligand for NKp30, was reported (54). Very recently, Deng et al. demonstrated that shedding of the NKG2D ligand MULT1 results in NK cell activation and increased surface expression of NKG2D (55). As suggested by the authors, the differential affinity of MULT1 (high-affinity NKG2D ligand) and MICA/B (low-affinity NKG2D ligand) for NKG2D may explain this discrepancy. Recently, Nanbakhsh et al. reported that the induction of c-myc in leukemic cells resistant to cytarabine resulted in up-regulation of NKG2D ligands (56). Since deregulated expression of c-myc is associated with many cancers in human, it raises the question of the expression of NK cell-activating ligands on c-myc-altered solid tumors. We and others have also provided evidence indicating a role of HIF factors in tumor resistance to NK cell-mediated lysis, which is further detailed in Section “Consequences of Hypoxia-Induced HIF Stabilization on NK Cell Functions.”

## Consequences of Hypoxia-Induced HIF Stabilization on NK Cell Functions

Microenvironmental hypoxia is a prominent feature of solid tumors and is involved in fostering the neoplastic process and in the modulation of immune reactivity (57). It results from inadequacies between the tumor microcirculation and the oxygen demands of the growing tumor mass, which leads to a lowering of oxygen partial pressure and a metabolic switch toward glycolysis (58). Tumor hypoxia is a negative prognostic and predictive factor due to many effects on the selection of hypoxia-surviving clones (59), activation of the expression of genes involved in apoptosis inhibition (60), angiogenesis (61), invasiveness and metastasis (62), epithelial-to-mesenchymal transition (63), and loss of genomic stability (64). Accumulating evidences indicate that tumor hypoxia is also involved in the loss of immune reactivity either by decreasing tumor cell sensitivity to cytotoxic effectors or by promoting immunosuppressive mechanisms (57).

### Cellular adaptation to hypoxia through hypoxia-inducible factors

Cells adapt to hypoxic microenvironment by regulation of hypoxia-inducible family of transcription factors (HIFs). This family comprises three members: HIF-1, HIF-2, and HIF-3. HIF-1 is a heterodimeric protein composed of a constitutively expressed β-subunit and an O_2_-regulated α-subunit. In the presence of O_2_, HIF-1α is hydroxylated on proline residue 402 and/or 564 by prolylhydroxylase domain protein 2 (PHD2), resulting in its interaction with the von Hippel-Lindau (VHL) tumor suppressor protein, which recruits an E3 ubiquitin-protein ligase that eventually catalyzes poly-ubiquitination of HIF-1α, thereby targeting it for proteasomal degradation (1). Under hypoxic conditions, hydroxylation is inhibited and HIF-1α rapidly accumulates, dimerizes with HIF-β, binds to the core DNA-binding sequence 50-RCGTG-30 [R being a purine base (adenine or guanine)] in the promoter region of target genes, recruits coactivators, and activates transcription (65). In addition, oxygen-dependent hydroxylation of asparagine-803 by factor inhibiting HIF-1 (FIH-1) blocks the interaction of HIF-1α with the coactivators P300/CBP under normoxic conditions, resulting in suppression of HIF transcriptional activity (66–68). Similar to HIF-1α, HIF-2α is also regulated by oxygen-dependent hydroxylation. HIF-1α and HIF-2α are structurally similar in their DNA-binding regions and dimerization domains but differ in their transactivation domains. Consistently, they share many target genes, but each one also regulates a unique set of genes (69). HIF-3α lacks the transactivation domain and may function as an inhibitor of HIF-1α and HIF-2α (70).

### Hypoxic and pseudo-hypoxic tumor cells are resistant to NK cell-mediated killing

It is now well established that the hypoxic tumor microenvironment favors the emergence of tumor variants with increased metastatic and invasive potential and alters immune reactivity as well (57).

Fink and colleagues reported the inhibition of NK cell cytotoxicity toward liver tumor cell lines under hypoxic conditions, suggesting for the first time that hypoxia is able to confer tumor resistance to NK cell-mediated cytotoxicity (71). Another study demonstrated that hypoxia decreased the expression of MICA (a NKG2D ligand) on osteosarcoma cell surface with a consistent decrease in the susceptibility of these cells to NK cell-mediated cytotoxicity (72). Consistently, HIF-1α knockdown using small interfering RNA increased the expression of cell surface MICA and concomitantly increased the level of soluble MICA. HIF-1α was also found to be inversely correlated with MIC gene expression, indicating that hypoxia was involved in the inhibition of NK cell reactivity toward tumor cells. Recently, we showed that hypoxia-induced autophagy in tumor cells mediated resistance to CTL (73). In this context, Baginska et al. demonstrated that hypoxia-induced autophagy in tumor cells was also involved in tumor resistance to NK cells via granzyme B degradation in autophagosomes (74).

In the particular context of renal cancer, hypoxic signaling is frequently constitutively active owing to the majority of renal cancers presenting with clear cell carcinoma (ccRCC) histology (75), which is usually associated with mutational or functional inactivation of the *VHL* gene (76). The VHL pathway targets the hypoxia-inducible factors (HIFs) family of transcription factors, in particular HIF-1α and HIF-2α, for ubiquitin-mediated degradation via the proteasome (77). Consequently, VHL inactivation leads to constitutive stabilization of HIFs, a process known as pseudo-hypoxia, and increased expression of HIF target genes. Our group has recently shown that, in VHL-mutated ccRCC cells, HIF-2 stabilization caused by mutated VHL induces up-regulation of ITPR1 which is involved in ccRCC resistance to NK cells (78). NK cells were found to induce a contact-dependent autophagy in ccRCC cells that was dependent on ITPR1 expression in tumor cells. Blocking ITPR1 expression in ccRCC cells inhibited NK cell-induced autophagy and suppressed ccRCC resistance to NK cells.

On the contrary, in non-tumoral cells, Luo and colleagues demonstrated that HIF-1α overexpression in HK-2 cells induces MICA expression and enhances NK cell cytotoxicity toward target cells as well as IFNγ secretion by NK cells (79). Antibody blocking experiments using anti-MICA mAb were able to down-regulate NK cell-mediated killing and IFNγ secretion toward HIF-1α-overexpressing HK-2 cells confirming the involvement of MICA in the increased NK cell reactivity.

### Hypoxia inhibits NK cell functions via HIfs

The specific role of hypoxia and HIFs on NK cells is not well studied.

Balsamo and colleagues showed that NK cells adapt to a hypoxic environment by up-regulating HIF-1α. They demonstrated that, under hypoxia, NK cells lose their ability to up-regulate the surface expression of the major activating NK-cell receptors (NKp46, NKp30, NKp44, and NKG2D) in response to IL-2 or other activating cytokines (including IL-15, IL-12, and IL-21). These altered phenotypic features correlated with reduced responses to activating signals, resulting in impaired capability of killing infected or tumor target cells. However, hypoxia does not significantly alter the surface density and the triggering function of the Fc-γ receptor CD16, thus allowing NK cells to maintain their capability of killing target cells via antibody-dependent cellular cytotoxicity (80).

Hypoxic primary tumors were shown to provide cytokines and growth factors capable of creating a pre-metastatic niche and a reduction of the cytotoxic functions of NK cells. In fact, Sceneay et al. reported that injection of mice with hypoxic mammary tumor cells resulted in increased CD11b^+^/Ly6C^med^/Ly6G^+^ myeloid and CD3^−^/NK1.1^+^ immune cell lineages infiltration into the lung and led to increased metastatic burden in mammary and melanoma experimental metastasis models (81). The cytotoxicity of NK cells was significantly decreased, resulting in a reduced antitumor response that allowed metastasis formation in secondary organs to an extent similar to that observed following depletion of NK cells. Sarkar and colleagues confirmed that hypoxia reduced NK cell killing of multiple myeloma cell lines (82). They showed that hypoxia significantly decreased expression of the activating receptor NKG2D by NK cells and of intracellular granzyme B and perforin. Whether HIF factors were able to directly regulate the expression of granzymes genes is not documented, but perforin has been reported not to be a direct target gene of HIF-1 (83).

Despite detailed description of the detrimental effects of hypoxia on NK-cell responses, the underlying molecular mechanisms remain unclear. In particular, whether HIF or other hypoxia-related factors are able to directly control NK cell receptor expression remain to be clarified.

### Indirect consequences of hypoxic stress on NK cell cytotoxic functions

Despite the direct consequences of hypoxic stress on NK cells, intratumoral hypoxia is also involved in increased tumor infiltration by Treg and MDSC and in M2-polarization (57), which are cellular subsets that negatively regulate NK cell lytic functions (see Cell-Mediated Immune Suppression Toward NK Cells in the Tumor Microenvironment). Hypoxic stress is also involved in increased expression and secretion by tumor cells of NK cell-inhibiting cytokines such as TGF-β (84, 85). Of note, NK cell adhesion on hypoxic endothelial cells was reported to be not altered (86), but NK cell infiltration into hypoxic tumors has not been extensively studied.

## NK Cell: A Role in Tumor Immunoediting

The major focus of immunotherapy approaches has been enhancing the effectiveness of host antitumor immunity. However, while accumulating evidences indicate that tumor microenvironment might evade the innate host immune response to ensure tumor development and survival (58, 87), NK cells have also been reported to play a role in the selection of tumor-resistant and tumor-tolerant cells and therefore to shape tumor microenvironment (88). While the mechanisms of CTL-induced tumor editing are well known (89), only limited knowledge on how NK cells induce tumor editing is available. In this regard, the involvement of NK cells in immune editing has been studied in relation to NKG2D and DNAM1 (90, 91). Guillerey and Smyth have elegantly demonstrated the NK cell activity in the cancer immune editing process with particular emphasis on the elimination and escape phases (92). NK cells have been also shown to kill immature DC because of their low amount of surface human leukocyte antigen (HLA) class I molecules (33) and therefore impact the quality of adaptive immune response. In this regard, Ghadially et al. have reported data indicating that in the absence of NKp46, graft-versus-host disease (GVHD) is greatly exacerbated, resulting in rapid mortality of the transplanted animals (93). Furthermore, these authors have demonstrated that the exacerbated GVHD is the result of an altered ability of immune cells to respond to stimulation by immature DCs (94). Buchser et al. have shown that classic cytolytic cells, including NK cells, can often promote survival and autophagy in target cells (95). These authors provided evidence indicating that NK cells are a primary mediator of autophagy in tumor target cells by a mechanism involving cytokines (IL-10, IL-2, IFNγ) and that cell-to-cell contact strongly enhanced lymphocyte-mediated autophagy. The authors suggested that the NK cell-mediated autophagy promotes cancer cell survival and may represent an important target for development of novel therapies.

## NK Cell-Based Immunotherapies in the Context of Tumor Microenvironment Complexity and Heterogeneity

Natural killer cell-based immunotherapies have the advantage of circumventing antigen recognition restriction since NK cells do not need antigen recognition to kill tumor targets. In this context, NK cell infusion has been useful in leukemic patients, probably due to their primary location being the blood. Indeed, most of hematological malignancies display an autologous NK cell deficiency specifically in myeloid diseases. Autologous NK cells do not control acute myeloid leukemia (AML) blasts and several mechanisms have been hypothesized: down-regulation of the ligands for NK-cell activating receptors or up-regulation of NK cell inhibitory receptors (96). Allogeneic NK cells do not bear this deficiency and have demonstrated their ability to kill AML blasts targets. In this context, the killer Ig-like receptor (KIR)-ligand mismatch is considered fundamental for their antitumor effects (97, 98, 99). Modulating the immune reconstitution following allogeneic transplantation with NK cells is a potential powerful tool to increase the graft versus leukemia (GvL) effect against AML blasts and tumor cells (100). NK cells do recover early following allogeneic transplantation and exert cytotoxicity through MHC unrestricted killing. High numbers of allogeneic circulating NK cells improved remission duration in patients with leukemia and consolidate engraftment following haploidentical transplants (101).

On the other hand, the therapeutic potential of NK cells in solid tumors is not yet clearly established. However, pre-clinical studies support the antitumor activity of NK cells against solid tumors (102). Phase I and phase II clinical trials based on adoptive transfer of irradiated NK cell lines or allogeneic NK cells have been made in breast, ovarian, melanoma, and renal cancer patients (98, 103). These trials revealed mild and transient toxicities following NK-92 infusion and some severe syndromes following allogeneic NK cell administration. Further studies are still needed to increase NK cell persistence and expansion.

Moreover, producing sufficient amounts of allogeneic NK cells for clinical applications remain a technical challenge in cell therapy programs despite their useful and safe infusion 10 years ago (104). Dampening negative regulators of NK cell lytic functions should also be explored, in particular in the context of solid tumors. Strategies aimed at inhibiting NK cell suppressors such as TGF-β, expansion of immunosuppressive cells, and expression of inhibitory checkpoints should be considered. In particular, targeting HIF-1α by antisense plasmid in xenografted mice led to NK cell-dependent tumor rejection (105). Various anticancer drugs have been shown to inhibit HIFs (106, 107). We believe that pharmacologic manipulation of hypoxic signaling will result in increased target killing by effector cells and in general improving of antitumoral immunotherapy. Whether the suppression of hypoxia may be a promising strategy that is selective for facilitating immunotherapeutic efficacy in cancer patients is at present investigated. Nevertheless, a better understanding of functionally distinct KIR or NK cell receptor subsets within NK cell population is still needed for designing optimal immunotherapy based on NK cell administration or reactivation.

## Conclusion

During the last few years, cancer immunotherapy has emerged as a safe and effective alternative to cancers that do not respond to classical treatments including those types with high aggressiveness. New immune modulators like cytokines, blockers of CTLA4/CD28 and PD-1/PD-L1 interactions, or adoptive cell therapy have been developed and approved to treat solid tumors and hematological malignant diseases. In these scenarios, cytotoxic lymphocytes mainly CTLs and NK cells are the ultimate responsible for killing the cancer cells and eradicating the tumor. Many mechanisms have been proposed for the functional inactivation of tumor-associated NK cells. Thus, the definition of tumor microenvironment-related immunosuppressive factors, along with the identification of new classes of tissue-residing NK cell-like innate lymphoid cells, represents key issues to design effective NK-cell-based therapies for solid tumors.

## Conflict of Interest Statement

The authors declare that the research was conducted in the absence of any commercial or financial relationships that could be construed as a potential conflict of interest.
